# MDM2 is implicated in high‐glucose‐induced podocyte mitotic catastrophe *via* Notch1 signalling

**DOI:** 10.1111/jcmm.13253

**Published:** 2017-06-23

**Authors:** Hui Tang, Chun‐Tao Lei, Chen Ye, Pan Gao, Cheng Wan, Shan Chen, Fang‐Fang He, Yu‐Mei Wang, Hua Su, Chun Zhang

**Affiliations:** ^1^ Department of Nephrology Union Hospital Tongji Medical College Huazhong University of Science and Technology Wuhan China

**Keywords:** MDM2, Notch1, podocytes, mitotic catastrophe

## Abstract

Podocyte injury and depletion are essential events involved in the pathogenesis of diabetic nephropathy (DN). As a terminally differentiated cell, podocyte is restricted in ‘post‐mitosis’ state and unable to regenerate. Re‐entering mitotic phase will cause podocyte disastrous death which is defined as mitotic catastrophe (MC). Murine double minute 2 (MDM2), a cell cycle regulator, is widely expressed in renal resident cells including podocytes. Here, we explore whether MDM2 is involved in podocyte MC during hyperglycaemia. We found aberrant mitotic podocytes with multi‐nucleation in DN patients. *In vitro*, cultured podocytes treated by high glucose (HG) also showed an up‐regulation of mitotic markers and abnormal mitotic status, accompanied by elevated expression of MDM2. HG exposure forced podocytes to enter into S phase and bypass G2/M checkpoint with enhanced expression of Ki67, cyclin B1, Aurora B and p‐H3. Genetic deletion of MDM2 partly reversed HG‐induced mitotic phase re‐entering of podocytes. Moreover, HG‐induced podocyte injury was alleviated by MDM2 knocking down but not by nutlin‐3a, an inhibitor of MDM2‐p53 interaction. Interestingly, knocking down MDM2 or MDM2 overexpression showed inhibition or activation of Notch1 signalling, respectively. In addition, genetic silencing of Notch1 prevented HG‐mediated podocyte MC. In conclusion, high glucose up‐regulates MDM2 expression and leads to podocyte MC. Notch1 signalling is an essential downstream pathway of MDM2 in mediating HG‐induced MC in podocytes.

## Introduction

Due to the growing prevalence of diabetes mellitus (DM) in worldwide, the most common microvascular complication of DM–diabetic nephropathy (DN) has become a dominant cause of end‐stage renal disease (ESRD) 1. DN is characterized by aggravating albuminuria and gradually loss of renal function 2. Histological alternations of DN include the hyperplasia of the mesangial cells, deposition of extracellular matrix (ECM) in glomerular mesangium, thickness of basement membrane in glomeruli and tubules and injury of the endothelial cells and podocytes 3. During the past decade, the pathophysiology of DN changes from a ‘mesangial cell‐central theory’ to a ‘podocyte‐central theory’^4^. Based on recent studies, podocyte injury and loss are considered as the early and critical event in DN development 5.

Podocytes are highly specialized cells lining the outer surface of the glomerular basement membrane and interdigitate with neighbouring podocytes to form the slit diaphragm. Maintaining its sophisticated structural elements including the abundant actin cytoskeleton and arborized appearance with primary, secondary and final foot process is essential for the integrity of the filter barrier. Various of insults can trigger podocyte injury resulting in effacement of the foot process, detachment from the basement membrane and/or podocyte death 6. Numerous human and experimental studies have demonstrated that the podocyte number or density was diminished during DN. Reduced podocyte number is also a predictor of DN progression 7. But the mechanism mediating podocyte loss is still unclarified 8. Although most of the *in vitro* and experimental animal studies demonstrated that cultured podocyte would undergo apoptosis when exposed to high glucose (HG), the conclusion was mostly based on the caspase activity or TUNNEL study 9, 10, but not the direct evidence of podocyte apoptosis insight 11, 12. On the other hand, a newly defined type of podocyte death called mitotic catastrophe (MC) was recently found in several types of glomerulonephritis patients. These podocytes were presented as double‐nuclei, multi‐nuclei, micronuclei cells or cells with aberrant mitotic spindle 13, 14, 15. This finding provides a new vision to look at podocyte loss in glomerular diseases. But the role of podocyte MC in DN has not been greatly elucidated.

Murine double minute 2 (MDM2) is a well‐known oncogene and an E3 ubiquitin‐protein ligase. It exerts multiple functions through p53‐dependent or p53‐independent pathway in plenty of pathophysiology processes 16. MDM2 inhibits the p53‐dependent cell cycle arrest and programmed cell death 17. This mechanism promotes cell proliferation because it allows cells with significant DNA damage to complete mitosis 18. Furthermore, MDM2 has a p53‐independent role in Notch1 signalling, which correlates to apoptosis inhibition and cell proliferation 19. A study of adriamycin nephropathy mouse model found that MDM2 antagonist nutlin‐3a alleviated the progression of glomerular disorders by directly protecting from mitosis‐related podocyte loss 13. But the role of MDM2 in podocyte cell cycle control in DN has not been well elucidated.

In this study, we investigated the ultrastructure of podocytes in DN patients, and the role of MDM2 in cell cycle control in high‐glucose‐treated human podocytes. We found aberrant mitotic podocytes in DN patients and cultured podocytes under HG stimulation. Our data also show that high glucose induced MDM2 enhancement as well as MC in podocytes. Genetic depletion of MDM2 largely abolished podocyte MC and subsequent podocyte injury. These data raise new thoughts about podocyte loss and injury in DN or other glomerular diseases.

## Materials and methods

### Human renal biopsy samples

DN patients diagnosed by clinical feature, laboratory tests and histology evidence were enrolled in this study. Renal biopsies were performed as part of a routine clinical diagnostic investigation in the Department of Nephrology, Union Hospital, Huazhong University of Science and Technology, Wuhan, China. Control group cases were obtained from para‐carcinoma renal tissue, matched with gender and age. Renal histology and electron microscopy were done by experienced laboratory technicians in the department. The use of parts of human renal biopsies for research purposes was approved by the Ethics Committee of Huazhong University of Science and Technology. All the procedures were performed according to the guidelines of National Institutes of Health (NIH).

### Cell culture, treatment and transfection

An immortalized human podocyte cell line was cultured and maintained as described previously 20. Cells were incubated at 33°C for proliferation in RPMI1640 medium supplemented with 10% foetal bovine serum, 100 U/ml penicillin and 100 U/ml streptomycin. After the cells reached at 70% confluence, they were transferred to 37°C for 10–14 days to allow differentiation before any experimental manipulations. Differentiated podocyte were exposed to media mixed 30 mM d‐glucose (Sigma‐Aldrich, St. Louis, MO USA) for indicated times. Moreover, 30 mM mannitol was mixed into culture medium to adjust for osmotic pressure of the control group. After treatment, the cell supernatant was collected to detect lactate dehydrogenase (LDH) activity using a commercial assay kit (Cayman Chemical, Ann Arbor, MI, USA). Nutlin‐3a (Selleck Chemicals, TX, USA), the selective small‐molecule antagonist of MDM2, was used to block the interaction between MDM2 and p53. 5 μM nutlin‐3a was mixed into the culture medium 2 hrs before HG treatment.

To knock down MDM2 expression in cultured podocytes, a recombinant lentivirus vector harbouring a short‐hairpin RNA sequence targeting human MDM2 (MDM2 shRNA) and control empty lentivirus (Scrambled shRNA) was constructed by JikaiGene (Shanghai, China). Recombinant adenovirus vector encoding human MDM2 sequence (Ad‐MDM2) and control empty adenovirus (Ad‐GFP) constructed by Vigene Biosciences (MD, USA) was used for MDM2 overexpression. Short‐interfering RNA targeting human NICD (NICD siRNA) and scrambled siRNA (Ribobio, Guangzhou, China) were constructed to knock down NICD expression. Differentiated podocytes were incubated with lentivirus or adenovirus or transfected with siRNA according to the manufacturer's instructions. 48 hrs after transfection, HG (30 mmol/l) was mixed into the medium for the corresponding time to conduct the following experiments.

### Immunofluorescence staining

Indirect immunofluorescence staining was performed with an established procedure 21. Briefly, differentiated podocytes cultured on clean coverslips were fixed in 4% paraformaldehyde in PBS for 30 min., permeabilized with 0.3% Triton X‐100 in PBS for 15 min. and then blocked with 5% BSA for 30 min. in 37°C. After that, the coverslips were incubated with mouse anti‐α‐tubulin antibody(1:100; Protein Tech Group, Chicago, IL, USA) or rabbit anti‐Ki67 antibody (1:100; Santa Cruz Biotechnology, Santa Cruz, CA, USA) overnight at 4°C. After PBS washing, these coverslips were incubated with Alexa‐488‐labelled secondary antibody at room temperature for 1 hr. Negative control was performed by replacing the primary antibodies with PBS. All the slides were counterstained with Hochest33258 (1:50; Santa Cruz Biotechnology) for 5 min. to show nuclei. The images were captured by a LSM 510 confocal microscope and LSM software (Carl Zeiss AG, Oberkochen, Germany) at identical microscopic settings and analysed using Image‐pro plus 6.0 software (Media Cybernetics, Rockville, MD, USA). The integrated optical density was used to evaluate relative amount of positive staining.

### F‐actin staining

Fluorescent staining of F‐actin was performed to show podocyte skeleton as described previously 22. Briefly, the cells were fixed in 4% paraformaldehyde for 15 min. at room temperature after washing, and then permeabilized for 15 min. with 0.3% Triton X‐100 in PBS. Cells were incubated with rhodamine–phalloidin (Invitrogen Corp., Carlsbad, CA, USA) for 15 min. at room temperature to stain F‐actin. The slides were examined by confocal laser scanning microscopy. One hundred cells were counted to calculate the ratio of cells retaining distinct F‐actin fibres in different groups.

### Cell cycle analysis

Cell cycle status was quantitatively determined by flow cytometry analysis. After indicated treatment, podocytes were trypsinized, washed with cold PBS and fixed in cold 70% ethanol overnight at 4°C. Cells were then incubated with propidium iodide (PI, 50 μg/ml) containing 0.1 mg/ml RNase A and 0.2% Triton X‐100 for 30 min. at 37°C. The DNA content was analysed by BD LSR (Becton Dickinson, Franklin Lakes, NJ, USA) to quantify the proportion of cells in each cell cycle phase, and 10,000 gated events were acquired per sample. Analysis of cell cycle distribution was performed with ModFit LT software (Verity Software House, Topsham, ME, USA).

### Western blot analysis

Immunoblotting was performed as previously described 21. In short, proteins from cultured cells were extracted with RIPA Lysis Buffer (Beyotime, Jiangsu, China). Protein concentration of cell lysates was measured by BCA Protein Assay kit (Bio‐Rad, Hercules, CA, USA). After boiling for 5 min. at 95°C in 5× loading buffer, an equal amount of protein (40 μg) was loaded onto an SDS‐polyacrylamide gel for electrophoresis and then transferred to PVDF membranes (Merck Millipore, Billerica, MA, USA). After blocking with 5%BSA for 1 hr, the membranes were incubated overnight at 4°C with the following primary antibodies: Mouse anti‐MDM2 antibody (1:500; Santa Cruz Biotechnology), Rabbit anti‐p53 (1:1000; Santa Cruz Biotechnology), Rabbit anti‐Aurora B antibody (1:1000; Cell Signaling Technology, Cambridge, MA, USA), Rabbit anti‐Phospho‐Histone H3 (ser10) antibody (1:1000; Cell Signaling Technology, Danvers, MA, USA), Rabbit anti‐SnoN antibody (1:500; Protein Tech Group), Rabbit anti‐NICD antibody (1:1000; Abcam, Cambridge, MA, USA), Rabbit anti‐Cyclin B1 (1:1000), Rabbit anti‐Cyclin D1 antibody (1:1000; Protein Tech Group), anti‐Cyclin E antibody (1:1000) and mouse anti‐β‐actin antibody (1:10,000; Santa Cruz Biotechnology). β‐Actin was used as an internal control. After incubation with appropriate secondary antibodies, the immunoreactive bands were detected by chemiluminescence methods and visualized on Kodak Omat X‐ray films. Densitometric analysis of the images obtained from X‐ray films was performed with the Image J software (NIH, Bethesda, MD, USA).

### Statistical analysis

All of the values were expressed as mean ± S.E.M. Significant differences among two groups were examined using the two‐tailed *T*‐test with GraphPad Prism (GraphPad Software, La Jolla, CA, USA). Statistical significance was defined as *P* < 0.05.

## Results

### Aberrant mitotic podocytes were presented in DN patients

**Figure 1:**
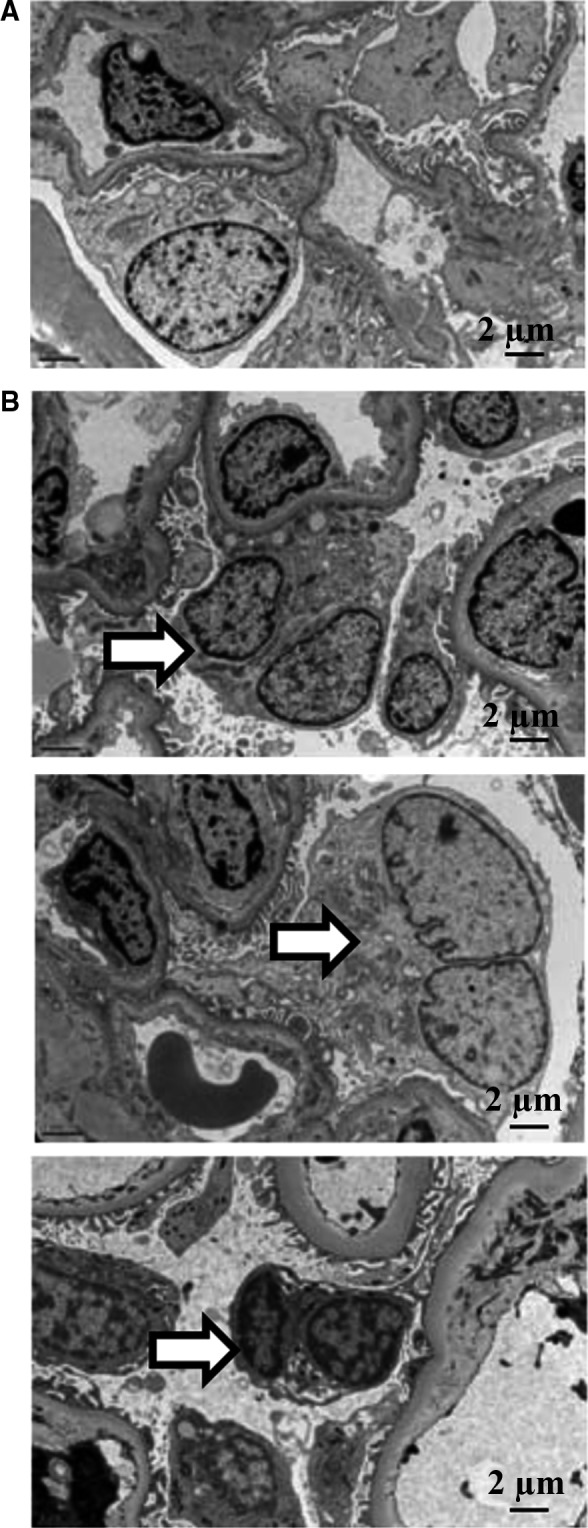
Aberrant mitotic podocytes were found in diabetic nephropathy (DN) patients. TEM examination shows the ultrastructure of podocyte from renal peritumoral tissues (**A**) and DN patients (**B**). Arrows indicate binuclear podocytes in renal tissue of DN patients. Original magnification: ×5000.

To observe the mitotic status of podocytes, we took advantage of scanning EM to show the ultrastructure of podocytes in DN patients. It was found that some of the podocytes in DN patients presented binuclear with dot‐like nuclear chromatin condensation and foot excess effacement, as shown in Figure 1. This is a direct evidence of podocytes in aberrant mitosis process in DN patients.

### HG exposure induced podocyte MC and increased the expression of MDM2

**Figure 2:**
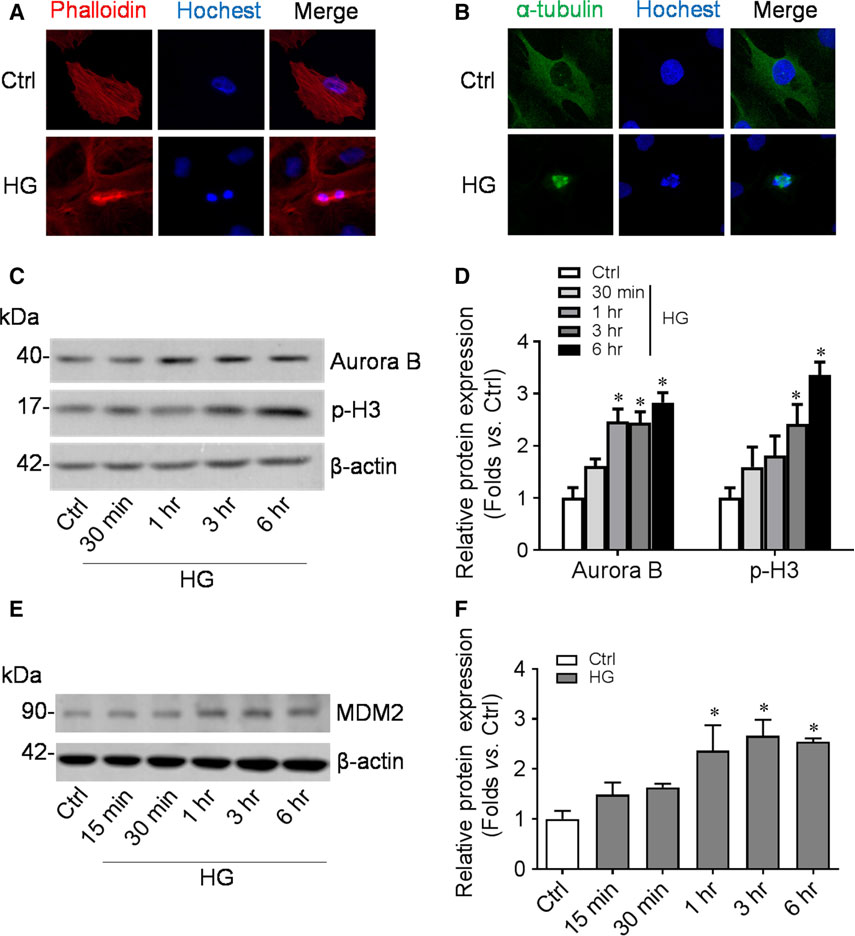
High glucose (HG) exposure induced podocyte mitotic catastrophe and increased the expression of MDM2. **A**‐**B:** Differentiated podocytes treated with HG were stained for F‐actin (Phalloidin, red), microtubule (α‐tubulin, green) or DNA (Hochest, blue). Original magnification: ×400; **C**‐**D**: Western blotting gel documents (**C**) and summarized data (**D**) showing the expression of Aurora B and p‐H3 in cultured podocytes under high‐glucose (HG) exposure for indicated time. *n* = 3. *P < 0.05 *vs*. Ctrl; **E**‐**F**: Western blotting gel documents (**E**) and summarized data (**F**) showing the expression of MDM2 in cultured podocytes under HG exposure for indicated time. *n* = 4. *P < 0.05 *vs*. Ctrl.

Morphologically, binuclear podocytes with disarranged F‐actin were presented under HG stimulation (Fig. 2A). Unlike bipolar distributed microtubules formed mitotic spindle in normal mitotic cells, HG‐treated podocytes presented muti‐polar mitotic spindle with irregular assigned chromosomes (Fig. 2B). To further explore whether podocyte could re‐enter into cell cycle and endure subsequent mitosis in diabetic status, we examined the protein level of mitotic marker Aurora B and p‐H3. A significant enhancement of Aurora B and p‐H3 expression was found in cultured podocytes after exposed to HG (Fig. 2C, D). Meanwhile, the expression of MDM2, a protein closely correlates to cell cycle control, was also in a similar time‐dependent manner (Fig. 2E, F).

### *In vitro*, genetic depletion of MDM2 partly reversed HG‐induced podocyte MC

**Figure 3:**
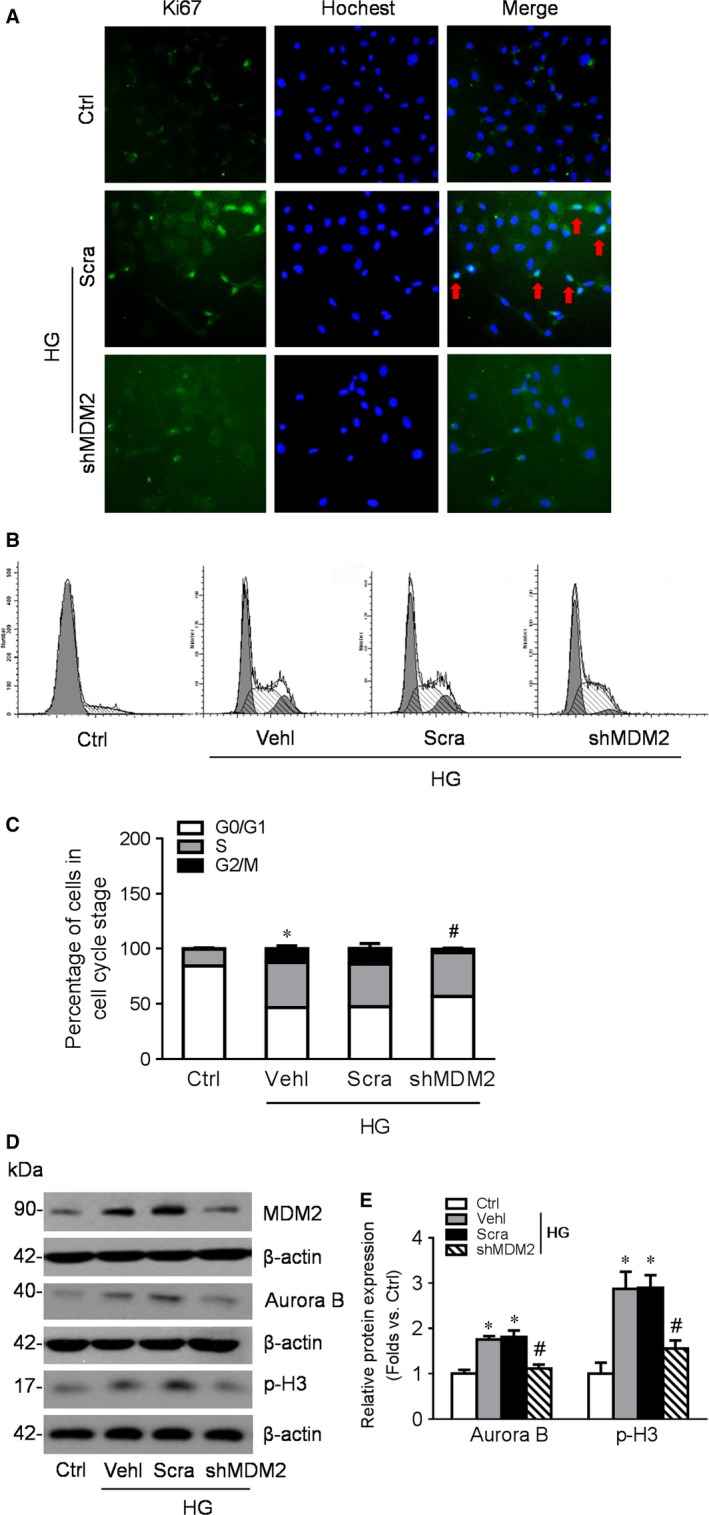
Genetic depletion of MDM2 partly reversed high glucose (HG)‐induced podocyte mitotic catastrophe. Differentiated podocytes were infected with lentivirus‐shRNA‐MDM2 or negative control lentivirus and subjected to HG treatment 48 hrs after infection. (**A**) IF staining showing the expression of Ki67 (green) and its colocalization with Hochest (blue) in podocytes of different groups (original magnification: ×200). **B**‐**C**: Cell cycle analysis by PI staining and flow cytometry of podocytes in different groups (**B**) and summarized data (**C**). *n* = 4. **D**‐**E**: Western blotting gel documents (**D**) and summarized data (**E**) showing the expression of MDM2, Aurora B and p‐H3 in podocytes of different groups. *n* = 5. *P < 0.05 *vs*. Ctrl, #*P* < 0.05 *vs*. HG + Scra. Ctrl: control; Vehl: vehicle; Scra: scrambled shRNA; shMDM2: MDM2 shRNA.

We genetically down‐regulated the expression of MDM2 in podocytes by lentivirus infection and examined the impact on podocyte cell cycle. Cultured podocytes in normal condition do not express Ki67, the marker of cell proliferation. But the number of Ki67‐positive podocyte significantly increased after high‐glucose stimulation, which was abolished by MDM2 genetic inhibition (Fig. 3A). Meanwhile, flow cytometry showed that a large number of podocytes in G0/G1 phase entered into S and G2/M phase after HG stimulation. However, MDM2 depletion significantly blocked high‐glucose‐induced G2/M phase entering, making majority of podocytes trapped in G1 and S phase (Fig. 3B, C). On the other hand, the up‐regulation of mitotic marker Aurora B and p‐H3 induced by HG was also prevented by MDM2 depletion (Fig. 3D, E).

### Differential expression of cyclins in podocytes with or without MDM2 deletion

**Figure 4:**
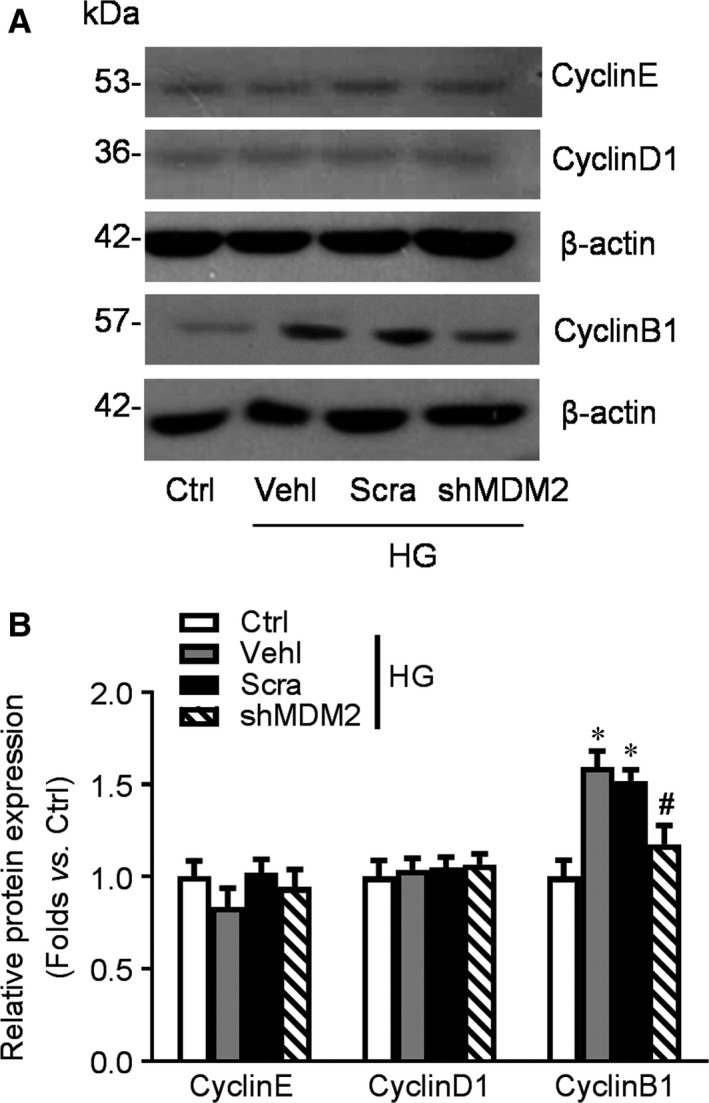
The expression of Cyclins in podocytes with or without MDM2 deletion. A‐B: Western blotting gel documents (**A**) and summarized data (**B**) showing the expression of Cyclin E, Cyclin D1 and Cyclin B1 in podocytes of different groups. *n* = 5. *P < 0.05 *vs*. Ctrl, #*P* < 0.05 *vs*. high glucose (HG) + Scra. Ctrl: control; Vehl: vehicle; Scra: scrambled shRNA; shMDM2: MDM2 shRNA.

To further explore the impact of MDM2 depletion on podocyte cell cycle, we examined the expression of cyclins that regulate different cell cycle phase. To our surprise, cyclin E and cyclin D1 expression were unchanged during high‐glucose exposure and MDM2 deletion. But cyclin B1 expression significantly increased by high‐glucose stimulation, which was in accordance with our previous study 20. Genetic knocking down of MDM2 partly reversed cyclin B1 enhancement induced by high glucose (Fig. 4A, B).

### Knocking down MDM2 alleviated HG‐induced podocyte injury

**Figure 5:**
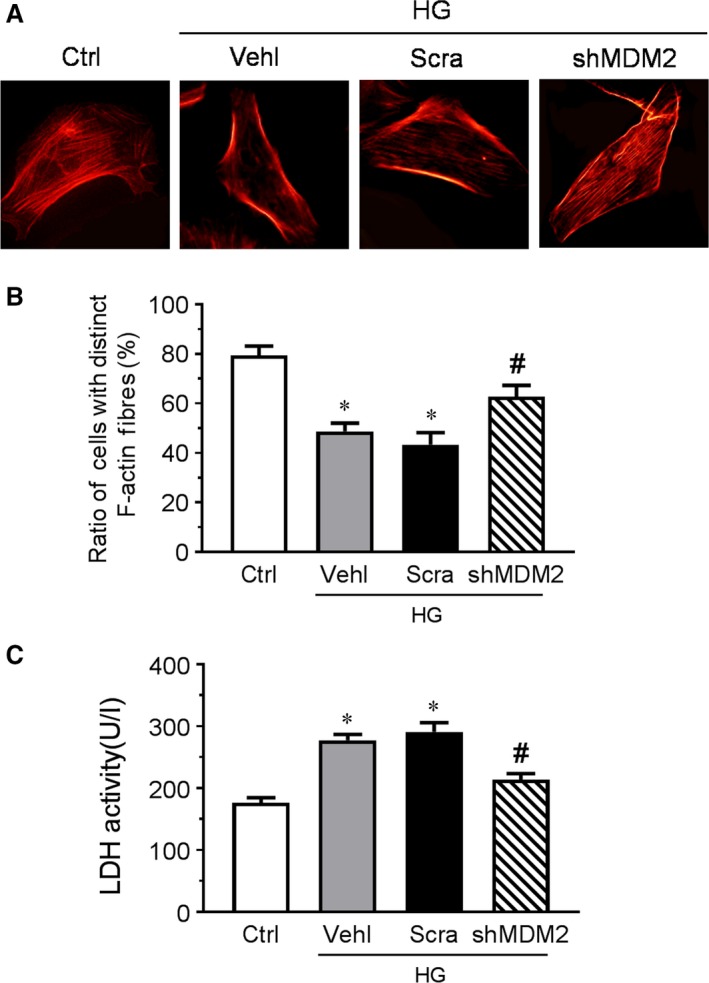
Knocking down MDM2 alleviates HG‐induced podocyte injury. (**A**) Microscopic images of F‐actin by rhodamine‐phalloidin staining (original magnification: ×400). (**B**) Summarized data from counting the cells with distinct, longitudinal F‐actin fibres. Scoring was determined from 100 podocytes on each slide. *n* = 5. (**C**) The LDH activity of podocytes in different groups. *n* = 5. *P < 0.05 *vs*. Ctrl, #*P* < 0.05 *vs*. HG + Scra. Ctrl: control; Vehl: vehicle; Scra: scrambled shRNA; shMDM2: MDM2 shRNA.

Podocytes re‐entering into cell cycle to regenerate will result in unsuccessful mitosis and podocyte death. The distinct, longitudinal F‐actin fibres in normal podocytes were replaced by disarrayed or diminished F‐actin fibres in high‐glucose‐treated podocytes, which was alleviated by MDM2 down‐regulation (Fig. 5A, B). Meanwhile, high‐glucose‐induced enhancement of LDH activity in culture medium was also largely abolished by the genetic depletion of MDM2 (Fig. 5C).

### Nutlin‐3a preserved p53 expression without influencing HG‐induced podocyte impairment

**Figure 6:**
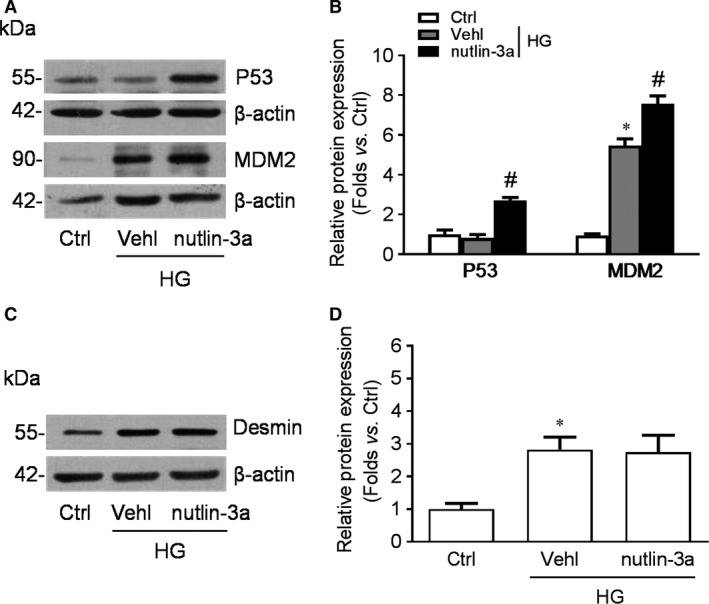
Nutlin‐3a preserved p53 expression without influencing high glucose (HG)‐induced podocyte impairment. **A**‐**D**: Cultured podocytes were pre‐treated by nutlin‐3a for 2 hrs before subjected to HG treatment. Western blotting gel documents (**A**) and summarized data (**B**) showing the expression of p53 and MDM2 in podocytes under HG exposure for 24 hrs. *n* = 4. Western blotting gel documents (**C**) and summarized data (**D**) showing the expression of Desmin in podocytes under HG exposure for 24 hrs. *n* = 3. *P < 0.05 *vs*. Ctrl, #*P* < 0.05 *vs*. Vehl + HG. Ctrl: control; Vehl: vehicle; nutlin‐3a: nutlin‐3a treatment.

MDM2 implicated in cell cycle control mainly through mediating p53 expression and translocation. Nutlin‐3a is a well‐known inhibitor of MDM2‐p53 interaction which abolishes MDM2 induced ubiquitination and degradation of p53. In our study, nutlin‐3a treatment up‐regulated the expression of p53 as well as MDM2 in cultured podocytes (Fig. 6A, B), which is consistent with previous studies. However, the enhancement of Desmin induced by high glucose could not be abolished by nutlin‐3a treatment (Fig. 6C, D).

### MDM2 mediated HG‐induced Notch1 activation in podocytes

**Figure 7:**
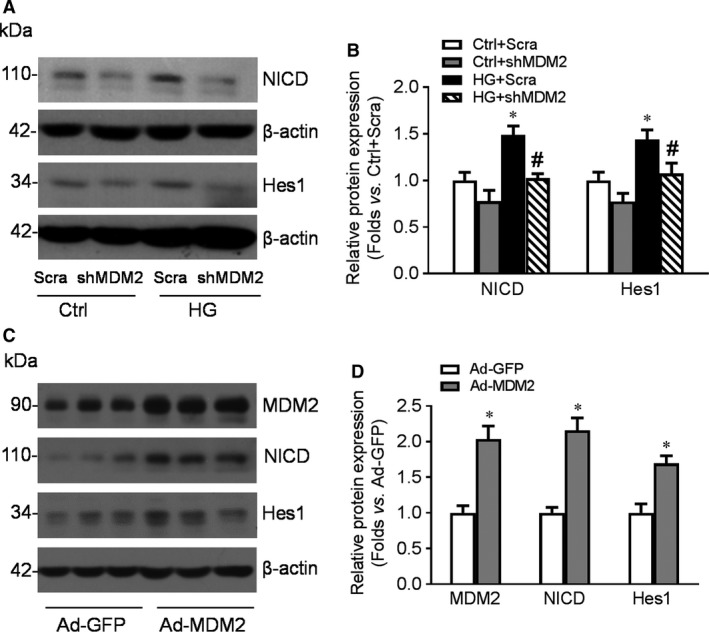
MDM2 mediated high glucose (HG)‐induced Notch1 activation in podocytes. **A**‐**B**: Cultured podocytes were infected with lentivirus‐shRNA‐MDM2 or negative control lentivirus and subjected to HG treatment 48 hrs after infection. Western blotting gel documents (**A**) and summarized data (**B**) showing the expression of NICD and Hes1 in podocytes of different groups. *n* = 6. *P < 0.05 *vs*. Ctrl + Scra, #*P* < 0.05 *vs*. HG + Scra. **C**‐**D**: Cultured podocytes were infected by Ad‐MDM2 or negative control Ad‐GFP. Western blotting gel documents (**C**) and summarized data (**D**) showing the expression of MDM2,NICD and Hes1 in podocytes of different groups. *n* = 6. *P < 0.05 *vs*. Ad‐GFP.

Activation of Notch1 pathway has been reported in cortex or podocytes of diabetic kidneys. In high‐glucose‐treated podocytes, we demonstrated a significantly up‐regulation of NICD, the intracellular fragment representing the activation of Notch1 pathway. MDM2 has been found promoting Notch1 activation through direct or indirect pathways in previous studies, but how it regulates Notch1 in DN has not been clarified. Our data showed that knocking down of MDM2 effectively abolished high‐glucose‐induced up‐regulation of NICD and the following activation of target gene Hes1 (Fig. 7A, B). Accordingly, overexpression of MDM2 also aggravated Notch1 activation and up‐regulated Hes1 expression (Fig. 7C, D).

### Knocking down Notch1 ameliorated HG‐induced podocyte MC

**Figure 8:**
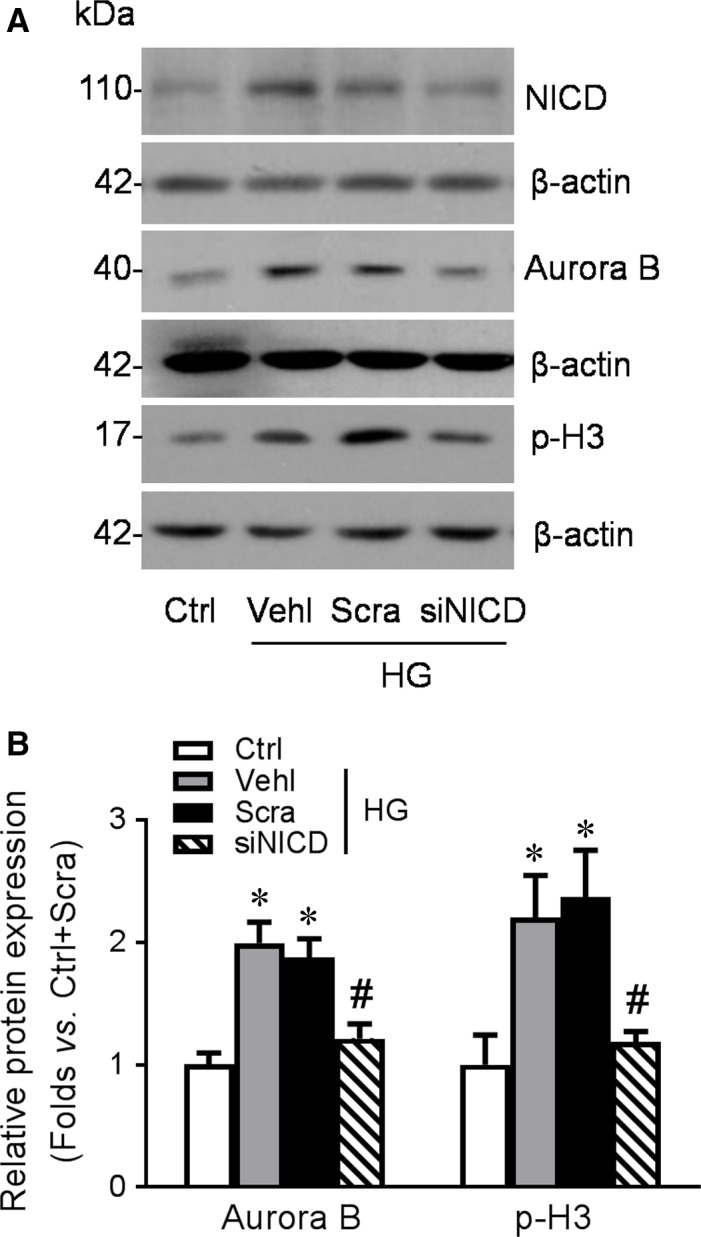
Knocking down Notch1 also ameliorates HG‐induced podocyte mitotic catastrophe (MC). **A**‐**B**: Cultured podocytes were transfected with NICD siRNA or scrambled siRNA and subjected to HG treatment 48 hrs after transfection. Western blotting gel documents (**A**) and summarized data (**B**) showing the expression of Aurora B and p‐H3 in podocytes of different groups. *n* = 3. *P < 0.05 *vs*. Ctrl, #*P* < 0.05 *vs*. HG + Scra. Ctrl: control; Vehl: vehicle; Scra: scrambled siRNA; siNICD: NICD siRNA.

To further confirm that Notch1 signalling pathway is the downstream of MDM2‐mediated podocyte MC, we genetically down‐regulated NICD expression in podocytes. The effective depletion of NICD resulting in reduction of high‐glucose‐induced up‐regulation of mitotic marker Aurora B and p‐H3 (Fig. 8A, B) shows a positive role of Notch1 signalling pathway in podocyte MC.

## Discussion

Podocyte dysfunction was considered as a consequence due to increased proteinuria rather than an early and critical event inciting DN until recent decades 4. Markedly reduced podocyte density is presented in early stage of patients with diabetes and experimental animal models, which strongly correlates with proteinuria level and DN progression 23, 24, 25. On the other hand, podocytes were also detected in the urine of diabetic patients with albuminuria which was another evidence for podocyte loss in DN 26. The exact aetiology for podocyte loss in diabetes remains speculative. Plenty of studies have suggested apoptosis as the mainly mechanism contributing to podocyte loss. However, it should be noted that although there were numerous data demonstrating podocyte apoptosis *in vitro* studies 9, 10, the definite evidence for a primary role of apoptosis in either human or experimental DN is scant, and apoptotic cell death is rarely seen in diabetic glomeruli *in situ* 11, 12. There were also studies showing that apoptosis may only be a late consequence due to detachment or other podocyte dysfunction rather than a predominate initiator in podocyte loss 27.

Several mechanisms of podocyte death besides apoptosis have been observed and described in various models of glomerular diseases, such as necrosis, autophagy, anoikis and others 28. Recent human and experimental studies in HIVAN, FSGS, MCD and IgAN have reported MC as a previous unrecognized cause of podocyte loss 13, 14. MC as a form of cell death was first observed in irradiated cancer tissues, characterized by abnormal nuclear configurations and spatial distribution of chromosomes and accompanied by changes in cell cycle checkpoint proteins, aurora kinases and cyclin B1‐dependent kinase Cdk1 levels 29, 30. Binucleated or multi‐nucleated cells with enlarged cytoplasm and nuclear changes are recognized features of aberrant podocyte mitosis 31. In our study, we first reported aberrant mitotic podocytes in the glomeruli of DN patients through EM. Some of these binucleated enlarged cells were still staying on the GBM with little‐to‐moderate foot excess effacement. Several of them could be floating in the Bowman space. Dot‐like nuclear chromatin condensation and cytoplasmic vacuoles could be seen in some of the mitotic podocytes.

Mature podocytes are terminal differentiated cells that quiescently arrested in the G0 phase. Unlike the podocyte precursor expressing plenty of Ki67, a marker of cell proliferation, matured podocyte exit cell cycle reduced the expression of Ki67, cyclin A and cyclin B1 but up‐regulated the expression of CDK inhibitors like p27 and p21 31. Studies have found absent p27, p57 and cyclin D1 expression and increased cyclin A, cyclin B1, cyclin E expression in human FSGS and adriamycin‐induced FSGS model 32, 33. Consistently in our data, cultured human podocytes were mainly arrest in G0 phase and lack of Ki67 expression. However, high‐glucose exposure induced the expression of Ki67 and cyclin B1, forcing majority of the podocytes to enter into S and G2/M phase, which might facilitate the MC process.

Studies from experimental membranous nephropathy have shown increased DNA synthesis in rat podocytes but no podocyte proliferation, indicating that matured podocytes have lost its regenerative property 34. Stressed podocytes may re‐enter into cell cycle but arrested in G1 or G2/M restriction point by CDK inhibitors, a process called podocyte hypertrophy 35. Mitogenic stimuli or DNA damage can force the podocytes to complete mitosis. However, podocytes need their actin cytoskeleton to maintain their sophisticated anatomical structure, leading to incomplete formation of mitotic spindles, aberrant chromosome segregation and/or podocyte detachment during mitosis. Podocytes cannot complete cytokinesis will result in aneuploid cells which are susceptible to death. Hence, podocyte mitosis is not a sign of podocyte regeneration but a pathologic mechanism of podocyte loss 15.

MDM2 is a well‐known oncogene and an E3‐ligase. It is critical for cell cycle control through mediating p53 ubiquitination and degradation and inhibiting the nuclear translocation of p53, which result in cell cycle arrest or abnormal mitosis. On the other hand, MDM2 interacts with numerous of cell cycle‐related molecules such as p73, Rb, E2F1 and MTBP 36, 37, 38. The p53‐independent interplay between MDM2 and these molecules exerts diverse impact on cell cycle. For example, MDM2 activates the expression of cyclin A and cyclin D1 and accelerates the cell into S phase, leading to abnormal reaction of the cell cycle checkpoint. Also, over expression of MDM2 abrogated ceramide‐mediated p21 (Cip1/Waf1) induction, G2 arrest and the late ensuing apoptosis. In acute kidney injury (AKI), MDM2 drives renal tubular regeneration by blocking p53‐mediated cell cycle arrest and apoptosis during post‐ischaemic 39. While in crescentic glomerulonephritis, MDM2 exerts pathogenic effects through p53‐independent NF‐kappa B activation induced intraglomerular inflammation and p53‐dependent parietal epithelial cell hyperplasia and crescent formation 40. But whether MDM2 is involved in cell cycle control or MC of podocyte in DN is uncertain. Our data showed that MDM2 expression was enhanced after a short period of high‐glucose stimulation. Genetic knocking down of MDM2 expression in podocytes cultured in high‐glucose medium reversed the increased number of cells positive for Ki67 staining, prevented podocytes from entering G2/M phase and partly blocks the activation of mitotic marker Aurora B and p‐H3 and cyclin B1. Consequently, podocyte injury marked by disarrangement of F‐actin and elevated LDH activity was also attenuated by MDM2 deletion. A recent study found that podocytes need MDM2 to maintain homoeostasis and long‐term survival *via* p53. Complete MDM2 knockout *in vivo* may result in proteinuria and glomerulosclerosis 41. It seems a proper level of MDM2 expression is essential for podocyte survival. Either up‐regulation or down‐regulation of MDM2 may result in podocyte impairment or death.

MDM2 ubiquitinates p53 for proteasomal degradation, suppresses p53 transcription and fosters nuclear export of p53 back to the cytoplasm 16. In this way, MDM2 prevents p53‐mediated cell cycle arrest, cell senescence and premature death 42. We speculated that MDM2 may promote podocyte MC by mediating p53 expression. Nutlin‐3a is a small chemical compound abolishes the interaction between MDM2 and p53, therefore preserving the function of p53. To our surprise, the usage of nutlin‐3a did not alleviate high‐glucose‐induced podocyte injury despite the remarkable up‐regulation of p53 level.

Previous studies have demonstrated that MDM2 activates the Notch signalling pathway by non‐degradative ubiquitination and synergizes with Notch1 to inhibit apoptosis and promote proliferation 19. Notch1 signalling is implicated in cell cycle re‐entry and progression 43 and has been widely reported its activation and involvement in various models of glomerular diseases 44, 45, 46, 47. Studies have demonstrated the up‐regulation of Notch1 pathway in diabetic mouse kidney and its involvement in high‐glucose‐induced podocyte apoptosis 48. In the present study, we also found a remarkable enhancement of NICD and its target gene Hes1 in high‐glucose‐treated podocytes. Genetic depletion or overexpression of MDM2 effectively down‐regulated or up‐regulated the expression of NICD and Hes1 respectively, indicating Notch1 pathway is the downstream of MDM2 in podocyte. Moreover, inhibiting Notch1 signalling by NICD siRNA greatly abolished high‐glucose‐induced activation of Aurora B and p‐H3, showing that Notch1 pathway activation contributes to HG‐induced podocyte MC.

In conclusion, here we provided the first evidence that under high‐glucose conditions, podocytes may re‐enter into cell cycle and approach to mitosis, but eventually result in MC. Up‐regulation of MDM2 induced by HG promotes podocytes to bypass G2/M checkpoint and contributes to podocyte MC. Furthermore, MDM2 is implicated in high‐glucose‐induced podocyte MC *via* activation of Notch1 signalling in a p53‐independent way.

## Authorship

Chun Zhang and Hua Su designed the research study; Hui Tang performed the research and wrote the paper; Chun‐Tao Lei, Chen Ye and Pan Gao helped to culture the podocyte; Cheng Wan, Shan Chen, Fang‐Fang He and Yu‐Mei Wang analysed the data.

## Conflict of interest

The authors declare that there is no conflict of interests regarding the publication of this manuscript.
